# Developments in Heliotherapy

**Published:** 1919-01-04

**Authors:** 


### DEVELOPMENTS IN HELIOTHERAPY.

WHAT struck first the visitor to the old-style hospital for surgical tuberculosis was a trophy of crutches in the entrance hall, put there to testify, *à la Lourdes*, to the cures wrought in, the wards beyond. Nowadays, or it would be better to say for the present (since everywhere in this country popular appeal is coming in fast), we consider this sort of thing playing to the gallery. But the visitor's attention is arrested just as much, at the new style sanatorium for tuberculous cripples, by the sight of the little inmates getting about out of doors, even in pretty cold weather, clad merely in bathing drawers and sunburn. This method of treatment—namely, heliotherapy—is the invention of Bernhard and Rollier. At Leysin, in the Alps, Rollier supplemented the open-air conservative treatment of non-pulmonary tubercle by exposing as much of the patient's body as possible to the action of the sun, which at high altitudes is very powerful all the year round. He and his followers contend that the benefit of this measure is much greater in cases that "tan" well, or develop a good deal of pigment or sunburn reaction, such as freckling. Moreover, a heavy coat of tan seems to prevent loss of heat, so that those in whom this is present can bear complete exposure in surprisingly low temperatures. And the questions arise, first, why does exposure to the sun help in scrofula and similar disease? secondly, how, if pigmentation is the reason, does the development of skin pigment antagonise the tuberculous process?

### VAGUE THEORIES.

Several theories have been advanced. Firstly there are those, such as recently Oehlecker, who deny any specific effect to the sun's rays, and say they merely act by general stimulation of the skin. The power of penetration of the ultra-violet rays is only one millimetre, whereas the tuberculous foci in bone and joint disease are deeply placed. By implication he denies that heavily pigmenting patients do especially well. Rollier believes that the pigment renders the solar rays "biologically effective"; and probably the presence of pigment can change rays of short wave-length into long. According to Jesionek, the pigment enters the blood vessels and is by them carried to the tuberculous focus, which in some way it neutralises. All these theories, it will be noticed, are somewhat vague. As against the first—namely, that of general stimulation—it can be urged that heliotherapy appears only of marked service in surgical tubercle; in consumptives the good effects, if any, are by no means so manifest.

### DEVELOPMENTS OF NATURE IMITATION.

It will readily occur to the reader that in many climates —that of the north of England, for instance—heliotherapy must necessarily lapse in winter. Abroad sundry attempts have therefore been made to improvise artificial sunlight. The first lamp used was that known as the mercury-quartz vapour lamp. That gave place to large carbon arc installations, and just lately special lamps constructed to imitate as nearly as possible the solar spectrum are being tried. At an annual meeting of sanatorium physicians in Berlin last June "Spectrosal lamps" by various makers, one at least with a metal filament, were mentioned. Up to the present artificial heliotherapy, as might be expected, seeing that it generally means indoor sessions of treatment, has been reckoned as inferior to natural. One worker has rhetorically estimated the difference as that between a Havana cigar and brown paper. It remains to be seen, however, what may be done by these later developments of Nature imitation. And even with the natural heliotherapy it is perhaps significant that the form of tuberculosis in which it is most esteemed is tuberculous peritonitis; for in children this disease is mostly of good prognosis, even without surgical measures; also it is described as of no avail in tuberculous ulceration of the intestines. Nevertheless, in the great improvement in results the conservative treatment of non-pulmonary tubercle has brought about, far surpassing those of the old operative *régime*, so that "surgical" tuberculosis is now a misnomer, a place must clearly be given to the action of sunlight on the human body.

